# Mechanistic Insights and Potential Use of Siderophores Producing Microbes in Rhizosphere for Mitigation of Stress in Plants Grown in Degraded Land

**DOI:** 10.3389/fmicb.2022.898979

**Published:** 2022-07-11

**Authors:** Pratiksha Singh, Prabhat K. Chauhan, Sudhir K. Upadhyay, Rajesh Kumar Singh, Padmanabh Dwivedi, Jing Wang, Devendra Jain, Mingguo Jiang

**Affiliations:** ^1^Guangxi Key Laboratory for Polysaccharide Materials and Modifications, School of Marine Sciences and Biotechnology, Guangxi Minzu University, Nanning, China; ^2^Department of Environmental Science, Veer Bahadur Singh Purvanchal University, Jaunpur, India; ^3^Guangxi Key Laboratory of Sugarcane Genetic Improvement, Sugarcane Research Institute, Guangxi Academy of Agricultural Sciences, Nanning, China; ^4^Department of Plant Physiology, Institute of Agricultural Sciences, Banaras Hindu University, Varanasi, India; ^5^Department of Molecular Biology and Biotechnology, Maharana Pratap University of Agriculture and Technology, Udaipur, India

**Keywords:** plant stress, siderophores, molecular mechanism, rhizospheric microbes, degraded land

## Abstract

Plant growth performance under a stressful environment, notably in the agriculture field, is directly correlated with the rapid growth of the human population, which triggers the pressure on crop productivity. Plants perceived many stresses owing to degraded land, which induces low plant productivity and, therefore, becomes a foremost concern for the future to face a situation of food scarcity. Land degradation is a very notable environmental issue at the local, regional, and global levels for agriculture. Land degradation generates global problems such as drought desertification, heavy metal contamination, and soil salinity, which pose challenges to achieving many UN Sustainable Development goals. The plant itself has a varied algorithm for the mitigation of stresses arising due to degraded land; the rhizospheric system of the plant has diverse modes and efficient mechanisms to cope with stress by numerous root-associated microbes. The suitable root-associated microbes and components of root exudate interplay against stress and build adaptation against stress-mediated mechanisms. The problem of iron-deficient soil is rising owing to increasing degraded land across the globe, which hampers plant growth productivity. Therefore, in the context to tackle these issues, the present review aims to identify plant-stress status owing to iron-deficient soil and its probable eco-friendly solution. Siderophores are well-recognized iron-chelating agents produced by numerous microbes and are associated with the rhizosphere. These siderophore-producing microbes are eco-friendly and sustainable agents, which may be managing plant stresses in the degraded land. The review also focuses on the molecular mechanisms of siderophores and their chemistry, cross-talk between plant root and siderophores-producing microbes to combat plant stress, and the utilization of siderophores in plant growth on degraded land.

## Introduction

The adequacy of the agricultural soil deteriorates owing to exposures to adverse environmental conditions such as salinity, drought, heavy metal stress, etc., which induces plant stress and reduces plant growth productivity which will trigger food scarcity in the future. The nutrient discrepancy in the plant is a common occurrence found in degraded land, and among different plant nutrients, iron is the essential ingredient for plant growth (Connorton et al., 2017). Rapid industrialization, urbanization, unsuitable land use (IPBES, 2018), fast agricultural practices (Keesstra et al., 2018), soil salinization (Edrisi et al., 2021), soil erosion (Paul et al., 2020a,b), invasion of alien species (Rai, 2021), poor governance management strategy (Gerber et al., 2014), overexploitation of natural resources, excessive mining (Upadhyay and Edrisi, 2021; Shakeel et al., 2022), etc., degrade more than 33% of global land resources through direct and indirect approaches (IPBES, 2018; Srinivasarao et al., 2021).

Iron deficiency is a communally observed phenomenon in CaCO_3_ (calcium carbonate)-rich desert soil at high pH (Alhendawi et al., 1997). The availability of iron in soil mostly depends on the range of pH, and the character trait of saline (pH 7.2–8.5) and alkaline (pH > 8.5) soil (Upadhyay and Chauhan, 2022) showed iron deficiency due to less solubility of iron at high pH (Mann et al., 2017). Flood and raised concentration of nitrates and phosphates (exogenous use of synthetic fertilizers) in soil reduces iron solubility, alters iron translocation, and induces iron deficiency in the plant (Becker and Asch, 2005).

The plant growth reduction mediated stress due to nutrient imbalance in degraded soil is a common phenomenon; the increase of degraded soil due to salinity, drought, heavy metal, etc. are reported by several workers (Ma et al., 2020; Upadhyay et al., 2021). Out of numerous detrimental factors, the lack of available iron for the plant is one of the major factors (Liliane and Charles, 2020). Several pieces of research demonstrated that plant growth-promoting rhizobacteria (PGPR) may be a promising tool for mitigating the adverse effect of degraded lands; for instance in saline soil (Upadhyay et al., 2009, 2011; Upadhyay and Singh, 2015) drought conditions (Igiehon et al., 2019), and heavy metal conditions (Bhojiya et al., 2022). Positive association between rhizosphere and microbes play a crucial role under iron-stressed degraded land owing to the secretion of iron-chelating compounds i.e., siderophore (Dertz et al., 2006). Plant root secretes siderophore to maintain the iron level for their metabolic and physiological activities in iron-stressed degraded soil, but is not attained at the perfect level (Herlihy et al., 2020). On other hand, siderophore-producing microbes (SPM) produce numerous iron-chelating compounds, which can cut short plant stress under iron-stressed soil. Siderophore-producing microbes produce siderophore and have activities of biofertilizers and bio-control for the plant; thus SPM acts as a signature for sustainable agriculture and is eco-friendly for crop production in degraded land Table 1 (Alam, 2014). Siderophore-producing microbes reduce Fe deficiency and enhance all physiological and biochemical processes of the plant under saline soil (Sultana et al., 2021), drought conditions (Kumar et al., 2016), and heavy metal-stressed soil (Hofmann et al., 2021). Siderophore also changes the oxidation states of heavy metals such as Cd, Cu, Ni, Pb, Zn, Th, U, and Pu and makes them less toxic (Schalk et al., 2011). Siderophore has a strong affinity for iron-chelating compounds, induces a bioremediation process, and enhances nutrient uptake and plant growth (Rajkumar et al., 2010). A bacterial strain like *Pseudomonas fluorescence* produces pyoverdines siderophore that increases mobility and reduces the toxicity of heavy metals under uranium mines (Edberg et al., 2010). Sharma and Johri (2003) isolated *Pseudomonas* from rhizospheric soil of *Zea mays* L., which produces a siderophore that showed a high affinity to chelate of Fe^3+^ ions. Ahmed and Holmstrom (2014) and Huo et al. (2021) reported that the use of SPM is a suitable approach for reducing plant stress on degraded soil. Bioavailability of iron reduces the saline soil condition which leads to iron deficiency in a plant, and thus the plant faces both salinity stress and iron deficiency (Sultana et al., 2021). To combat iron deficiency under saline conditions, Sultana et al. (2021) isolated four salt-tolerant plant-growth promoting bacteria from rice rhizosphere, *Bacillus aryabhattai* MS3, which showed maximum siderophore producing ability at 200 mM NaCl concentration than the control. The siderophore-producing ability of *B. aryabhattai* MS3 increased due to the activation of *entD* gene by salinity, and *entD* gene has to be responsible for siderophore biosynthesis (Sultana et al., 2021). *Streptomyces tendae* F4 reduces cadmium translocation from rhizosphere to plant in heavy metal polluted soil (Dimkpa et al., 2009). Similarly, Sadeghi et al. (2012) observed that the isolate C (*Streptomyces*) increased siderophore production in the presence of a high concentration of NaCl (300 mM), and also produced auxin, solubilized tricalcium phosphate. Inoculation of isolate C (*Streptomyces*) increased iron content in the shoot of wheat plants in saline soil (Sadeghi et al., 2012). Therefore, in this context, the present article aims to provide recent updates on plant mechanisms under iron-stressed degraded soil, nexus between plants siderophores and siderophore producing bacteria, and developing sustainable use of siderophore-producing bacteria for plant growth under degraded soil.

**Table 1:** Recent studies (2016–2021) showing the main effects on plants exerted by siderophore-producing rhizobacteria alone or in combination in degraded soil conditions.

**SPRB**	**Types of siderophore**	**Condition**	**Plants**	**Performances**	**References**
*Fluorescent pseudomonads*	Pyoverdines	Iron-Limited conditions	*Arabidopsis thaliana*	Potential for plant growth and increased immunity	Trapet et al. (2016)
*Bacillus* spp.	Catecholate and salicylate	Field experiments	Potato and banana	*Bacillus niabensis* (PT-32-1), *Bacillus subtilis* (SWI16b), *Bacillus subtilis* (HPC21) from Phototo rhizosphere induces plant growth and *Bacillus mojavensis* (JCEN3) inhibits the pathogens of wilting disease in banana	Kesaulya et al. (2018)
*Pantoeadispersa* MPJ9 *and Pseudomonas putida* MPJ2	Catecholate	Iron limiting condition	*Vigno radiata*	Both MPJ9 and MPJ2 increased 89.9 and 85.3% siderophore production, respectively, and enhances iron 100.3 ppm, 0.52 (g/g) protein, and 0.67 (g/g) carbohydrates content in *Vigna radiata* plant under pot experiment	Patel et al. (2018)
*Bacillus* MG214652 and *Aspergillus niger* MH844535	Catecholate and hydroxymate	Iron deficient condition	*Phaseolus vulgaris, Pisum sativum, Vivia faba and Alfa alfa*	*Bacillus* (MG214652) and *Aspergillus niger* (MH844535) are potential catecholate and hydroxymate types of siderophore producers, respectively that enhance plant nutrients and soil health and promote plant growth	Osman et al. (2018)
*Bacillus subtilis*	Endophytic siderophore	Drought condition	*Triticum aestivum*	Enhances the survivability and potential growth of wheat plant drought condition	Lastochkina et al. (2020)
*Streptomyces* sp. S29	Desferrioxamines and hydroxamate	Drought condition	*Lupinus oreophilus*	Desferrioxamines siderophore prevent from fungal disease while Hydroxamate types of siderophore enhance iron content	Jarmusch et al. (2020)
*Bacillus megaterium and Pantoeaallii*	Hydroxymate	Alkaline conditions	–	Highest iron-chelating ability was reported in *Bacillus megaterium* followed by *Bacillus subtilis and Azotobacter vinelandi*, respectively, at pH = 9, which indicates that these bacterial isolates can reduce iron deficiency in plant and mitigate chlorosis under saline soil	Ferreira et al. (2019)
*Bacillus subtilis and Rhizobium radiobacter*	Catecholate	Alkaline conditions			
*Azotobacter vinelandii*	Both satecholate and Hydroxymate	Alkaline conditions			
*Penicillum chrysogenum, Aspergillus sydowii* and *Aspergillus terreus*	Hydroxymate	Pot experiments	*Cymbidium aloifolium*	Enhances the nutrient uptake and resistance against plant pathogens in crops	Chowdappa et al. (2020)
*Dermacoccusbarathri* MT2.1T, *D.profundi* MT2.2T, and *D. nishinomiyaensis* DSM20448T	Catecholate and hydroxymate	Saline condition	*Lycopersicon esculentum*	Increased tomato seedling and plant growth	Rangseekaew et al. (2021)
*Bacillus subtilis* LSBS2	Bacillinbactin	Iron limiting condition	*Sesamum indicum*	Increased HCN, IAA, ammonia, and siderophore production that enhanced the nutrients including iron in sesame plant	Nithyapriya et al. (2021)
*Bacillus subtilis* MF497446 and *Pseudomonas korensis* MG209738	Hydroxamate	Green house and field condition	*Zea mays*	Significantly increases catalase (CAT), peroxidase (POX), and polyphenol oxidase (PPO) activities, plant chlorophyll and carotenoids that increase crop yields compared to control	Ghazy and El-Nahrawy (2021)
*Streptomyces ciscaucasicus* strain GS2	Ferrioxamines	*In vitro* condition	*Malus domestica*	Prevents the apple replant disease and enhances plant growth and yields	Armin et al. (2021)
*Pseudomonas fluorescens* SBW25	Hydroxymate	Iron-Limited conditions	*Brachypodium distachyon*	Phytosiderophore provides defense under stress conditions of plant growth	Boiteau et al. (2021)
*Bacillus subtilis*	Catecholate	Pot experiments	*Coriandrum sativum*	Significantly acts as a biofertilizer that enhances seed germination and plant growth	Kumari et al. (2021)
*Pseudomonads*	Pyoverdine	Field experiment	*Pisum sativum*	Enhances root and shoot length	Lurthy et al. (2020)
*Fluorescent Pseudomonas*	Hydroxymate	*In vitro* condition	*Zea mays*	Inhibits fungal pathogen *Fusarium oxysporum* and enhances the iron uptake in plants	Deori et al. (2018)
*Bacillus licheniformis* DS3	Hydroxymate	Field experiment	*Vigna mungo* (L.)	Biological agents that control several fungal pathogens like *Aspergillus niger, Alternaria solani, Fusarium solani*, and *Fusarium oxysporium*	Silpa et al. (2018)
*Pseudomonas* species	Hydroxymate	Field experiment	–	Siderophore acts as a biofertilizer	Joshi et al. (2018)
*Proteobacteria, Actinobacteria, Bacteroidetes*, and *Firmicutes*	Hydroxymate and catecholate	Spider cave and Lechuguilla cave	–	Acts as bioremediation agents	Duncan et al. (2021)
*Bacillus subtilis* (LSBS2)	Bacillibactin	Field experiment	*Arachis hypogaea*	Enhances the immunity of peanut plant	Latitha and Nithyapriya (2020)
*Pseudomonas* sp.	Hydroxymate and catecholate	Field experiment	*Cicer arietinum, Capsicum frutescens, Punica granatum*, and *Allium cepa*	Potential to increase plant growth	Parveen and Latha (2019)
*Pseudomonas furukawaii, Pseudomonas plecoglossicida, Pseudomonas alcaligenes, Pseudomonas oleovarans, Leclercia adecarboxylata, Citrobacter youngae, Enterobacter cloacae*	Hydroxymate and catecholate	Field experiment	*Phaseolus vulgaris, Helianthus, Triticum aestivum*, Oryza sativa	Antagonistic activities against different phytopathogens like *Rhizoctonia solani, Phythium* sp., *Fusarium oxysporum*	Khaing et al. (2021)
*Enterobacter* species, *Azotobacter* species, and *Pseudomonas* species	Hydroxymate and Catecholate	*In vitro* condition	*Gossypium hirsutum*	Potentially act as biocontrol agents against harmful plant pathogens	Patel and Minocheherhomji (2018)
*Pseudomonas citronellolis* strain SLP6	Hydroxamate	Salinity stress condition	*Helianthus annuus*	Significantly enhances chlorophyll content, antioxidant enzymes production, and plant growth	Silambarasan et al. (2020)
*Rhizobium* sp. strain R1	Catecholate	Drought	*Glycine max* L	Significantly enhances the soybean seed germination	Igiehon et al. (2019)

## Plant Stress Under Iron Deficient Degraded Land

**Figure 1:**
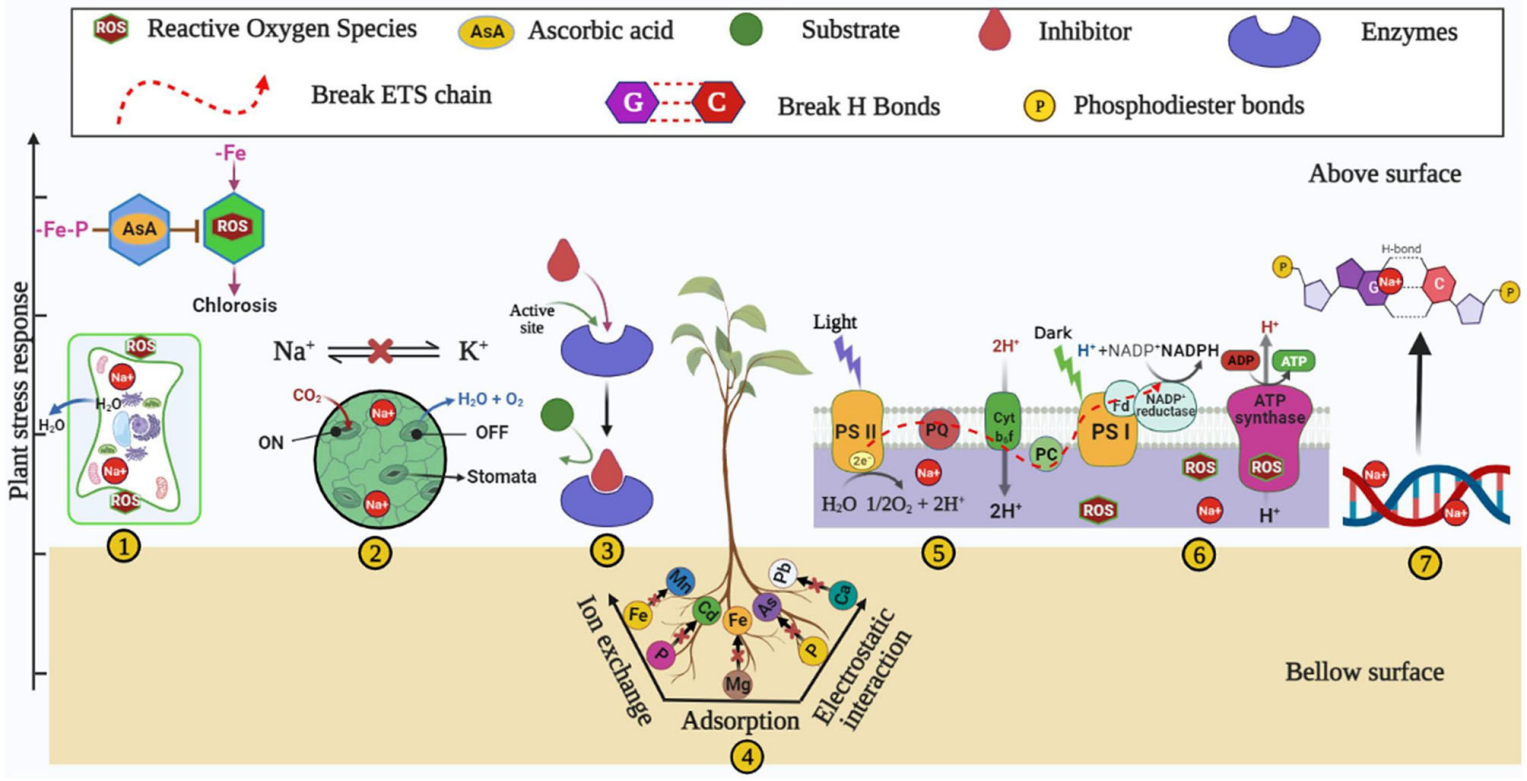
Plant stress responses such as (1) wilting and chlorosis, (2) altered stomatal activities, (3) inhibition of enzymatic activities (4) nutrient imbalance (5) increased ROS, (6) altered electron transport system, and (7) DNA damage under iron-deficient/degraded soil.

Soil degradation is a natural and anthropogenic phenomenon that reduces soil nutrients (Abiala et al., 2018; Upadhyay and Chauhan, 2019; Bhojiya et al., 2022; Shakeel et al., 2022), mediated by soil salinization (Qadir et al., 2014; Machado and Serralheiro, 2017; Abiala et al., 2018; Upadhyay and Chauhan, 2022), drought (Bartels and Sunkar, 2005), and heavy metals contamination (Paul et al., 2020b,c). The occurrence of an available form of Fe lacks in almost all types of soil (neutral, acidic, and alkaline) due to several factors such as soil pH, deposition of CaCO_3_, saline and desert conditions, etc. (Alhendawi et al., 1997). Degraded soil adversely impacts the growth and output of plants through an imbalance of many nutrients and metabolic pathways (Figure 1) and induces the unfavorable fitness of soil for plant growth. Despite several detrimental factors of degraded soil, the present review discusses iron homeostasis and its possible ability to meet plant sustainability. Iron deficiency hinders several metabolic and physiological aspects in plants and human beings. The crucial role of iron has been well-acknowledged for several redox reactions of different physiological mechanisms of plants like respiration- and photosynthesis-mediated electron transport systems. Iron also participates in several enzymatic activities such as peroxidase, catalase, cytochrome, oxidase, etc. (Tripathi et al., 2018). Also, Fe plays the role of a co-factor in the synthesis of many plant hormones like ethylene and ACC deaminase (Siedow, 1991). Iron plays a crucial role in chlorophyll biosynthesis by maintaining electron flow in CO_2_ fixation through (PS)-II-b6f/Rieske (PS)-I complex (Ermakova et al., 2019). Iron plays a remarkable co-factor in the electron transport chain of plant photo-system. In photosystem (PS)-I , iron is required to form three 4Fe-4S in clusters, Cytochrome-b6f (Cyt-b6f) requires iron for Rieske subunits as a cluster of 2Fe-2S (Fukuyama et al., 1980; Hurt and Hauska, 1981), and photosystem (PS)-II requires iron as a cofactor for cytochrome (Ben-Shem et al., 2003). Iron is essential for leghemoglobin and nitrogen-fixing machinery in the leguminous plant (Brear et al., 2013). The deficiency of Fe leads to several disorders in the plant by altering the redox and enzymatic reactions and shows primarily a symptom of wilting and chlorosis (Bashir et al., 2016), which leads to a lowering of plant growth and productivity (Figure 1). Several researchers reported that root growth of the plant is hindered under Fe deficient soil (Satbhai et al., 2017), altering the function of the gene responsible for iron uptake (Colangelo and Guerinot, 2004). Iron stress also triggers a plant's reactive oxygen species-mediated Fenton reaction (Tewari et al., 2013; Dumanovic et al., 2021). The Fenton reaction elaborates the interplay of Fe^2+^ and H_2_O_2_ (hydrogen peroxide) to generate hydroxyl radical (OH^*^), which is one of the reactive oxygen species (Kar and Chattopadhyaya, 2017).

Iron stress also leads to necrosis in tissue, blackening of roots, and an overall decrease in plant growth (Rai et al., 2021). Degraded soil due to salinity increases ionic and osmotic stress and reduces plant growth and productivity (Upadhyay et al., 2012; Orozco-Mosqueda et al., 2020). Ionic stress induces the influx of Na^+^ ions, resulting in the efflux of K^+^ ions in soil (Yang et al., 2009; Orozco-Mosqueda et al., 2020), while osmotic stress accumulates the NaCl concentration in the rhizospheric soil (Egamberdieva et al., 2019). Soil salinity induces nutrient imbalance (Upadhyay and Chauhan, 2022) and iron deficiency (Rabhi et al., 2007; Sultana et al., 2021). Both iron and NaCl stresses induce reactive oxygen species (ROS), which directly causes injury to the plant tissue (Rabhi et al., 2007; Jha and Subramanian, 2020; Kamran et al., 2020), and salinity damages the base and cross-correlation of double-stranded DNA (Santoyo and Strathern, 2008; Orozco-Mosqueda et al., 2020). More salinity and iron deficiency affect the morphological traits such as a decrease in root length, plant size, variety of leaves, flowering of plants (Rabhi et al., 2007; Kapoor and Srivastava, 2010; Mallahi et al., 2018), decrease in the plant's pigment chlorophyll content, resulting in reduced photosynthesis (Ashraf et al., 2017); hence poor plant growth reduces the crop productivity (Palaniyandi et al., 2014; Machado and Serralheiro, 2017).

Rapid changes in climatic conditions alter the cycle of atmospheric rain, precipitation, and biogeochemical cycle, leading to an increase in Fe deficient soil and degraded land, developing water-deficit soil environment, etc. (Morrissey and Guerinot, 2009; Lal, 2012; Fuentes et al., 2018; Sileshi et al., 2020). Therefore, drought stress is noticed at a global level (Takahashi et al., 2020), and a substantial decrease in plant growth and productivity has been observed under drought stress-mediated iron-deficient soil (Tripathi et al., 2018). Heavy metals are found in degraded land, which poses hazardous environmental stress that arises both naturally and anthropogenically (Wasi et al., 2012; Bernard et al., 2018). An increase in the concentration of heavy metals in the soil creates various problems for flora and fauna (Alengebawy et al., 2021). Leskova et al. (2017) reported that Fe deficiency is a common phenomenon in soil contaminated with heavy metals. In the purview to tackle these issues, it is, therefore, necessary to develop a sustainable approach that improves plant growth and productivity under iron-stressed/degraded soil. The following section of this review discusses the possible application of siderophores-producing bacteria for plant growth under iron-deficient soil.

## Siderophore-Producing Bacteria

Siderophore-producing rhizobacteria that promote plant growth were demonstrated by several researchers, for example, *Bacillus subtilis, B. licheniformis, B. coagulanse, B. circulance, Pseudomonas koreensis, P. fluroscence* (Ghazy and El-Nahrawy, 2021), *P. aeruginosa* (Subramanium and Sundaram, 2020; Singh et al., 2021a), *Pseudoalteromonas tetraodonis, Bacillus cereus, Psychrobacter pocilloporae, Micrococcus, aloeverae, Pseudomonas weihenstephanensis* (Sinha et al., 2019), *Pseudomonas* sp. (Singh et al., 2022), *Enterobacter genera, Bacillus*, and *Rhodococcus* (Sah and Singh, 2015), *Bacillus megaterium* (Singh et al., 2020a), *Pantoae cypripedii* (Singh et al., 2021b), *Kosakonia radicincitans* (Singh et al., 2020b), and *Pantoae dispersa* (Singh et al., 2021c).

The environmental conditions such as pH, temperature, nutrient sources, aerobic/anaerobic, etc. influence the production of bacterial siderephores. Sinha et al. (2019) isolated *Enterococcus casseliflavus* and *Psychrobacter piscatorii* from Kerguelen Islands, and *P. astetraodonis, B. cereus, P. pocilloporae, Micrococcus aloeverae*, and *P. weihenstephanesis* were isolated from Prydz Bay. Isolates from Prydz Bay-produced either hydroxamate or catecholate types of siderophores at 15–25°C and 8.5 pH. *Pseudomonas fluorescens* synthesizes pyoverdine type of siderophores that enrich the ferric iron as nutrients in *Solanum lycopersicum* plants enhancing photosynthetic pigments and biomass of the plant (Nagata et al., 2013); *B. subtilis* produces hexadentate triscatecholamide bacillibactin which has an affinity to chelates the iron (Dertz et al., 2006); *P. aeruginosa* and *P. fluorescens* are capable of producing siderophore that increases the rate of phytoextraction and phytoremediation of heavy metals (Braud et al., 2009a). Essen et al. (2007) reported that *Pseudomonas strtzeri* 36,651 produces ferrioxamine type of siderophore under both aerobic and anaerobic conditions, and non-sulfur bacterium *Rhodopseudomonas palustris* str. CGA009 produces two types of siderophore rhodopertobactin under both aerobic and anaerobic conditions (Baars et al., 2018).

## Siderophores: Chemistry and Mechanism

Siderophores facilitate several functions of plants such as respiration (Aznar and Dellagi, 2015), photosynthesis (Nagata et al., 2013), bioremediation (Saha et al., 2016), plant growth promotion (Yadav et al., 2011; Ghazy and El-Nahrawy, 2021), and phytoremediation of heavy metals (Kong and Glick, 2017; Leguizamo et al., 2017; Ustiatik et al., 2021). Siderophores are also produced by non-ribosomal peptides bonds (Hu and Xu, 2011) and multidentate iron-chelating compounds that solubilize and chelate organic and inorganic forms of compounds in soil (Singh et al., 2017). The term is derived from the Greek words *sidero* meaning “iron” and *phore* meaning “carriers” or iron-bearing compounds that uptake insoluble iron from different environmental sources (Nagoba and Vedpathak, 2011). Primarily siderophore-producing bacteria release iron-binding proteins, such as permeases and ATPases, that chelate the ferric iron (Fe^3+^) and transport Fe^3+^ ions in the cell membrane in gram-positive bacteria (Ahmed and Holmstrom, 2014). Gram-negative bacteria have a complex mechanism for the transportation of ferric iron (Fe^3+^) mediated by many enzymes, periplasmic binding proteins, outer membrane receptors, and cytoplasmic membrane proteins which make Fe^3+^ available for plant cells (Ahmed and Holmstrom, 2014; Schutze et al., 2015).

**Figure 2:**
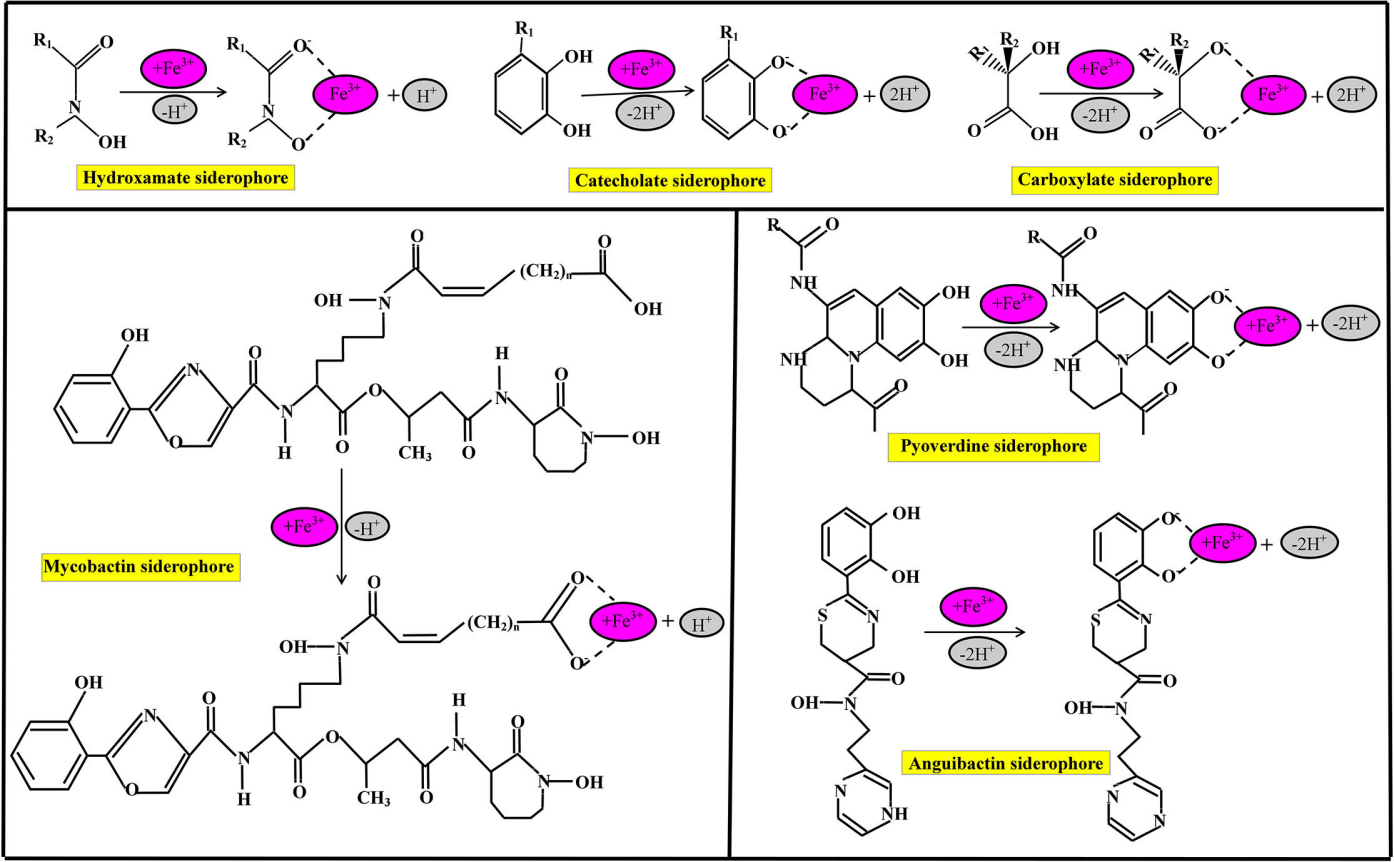
Chemical nature of commonly identified siderophores and their iron (Fe^3+^) chelating binding sites.

Siderophores are classified based on many criteria such as the source of siderophore, cyclic and linear structure of siderophore, and the chemical nature of functional groups of the siderophore, as shown in Figure 2. On the basis of functional groups, siderophores are classified as hydroxamate-type siderophore, catecholate-type siderophore, carboxyalate-type siderophore, and mixed ligand siderophore (Ito and Butler, 2005; Zawadzka et al., 2006; Butler and Theisen, 2010). Hydroxamate siderophores are a group of C(=O) N-(OH)R, where R is either amino acid or a derivative of amino acids, which contains two oxygen molecules to form bidentate ligand with iron ions; therefore, each siderophore is able to form hexadentate ligands, octahedral complex compounds with Fe^3+^ ions at a different range between 1,022 and 1,032 M^−1^ (Winkelmann, 2007). During the combination of hydroxamate with Fe^3+^ ions, hydroxamate functional group loses a proton from the hydroxylamine (-NOH) group to form a bidentate ligand (Fiestner et al., 1993).

Some bacterial species have the potential to hydroxamate siderophore production, including *P. aeruginosa*, which is able to produce pyoverdin hydroxamate type of siderophore under limited iron conditions (Meneely and Lamb, 2007). Catecholate, commonly known as phenolate (2, 3–dihydroxy benzoate) siderophore is an orthoisomer of three molecules of isomeric benzenediols (Sah and Singh, 2015). The functional group of catecholate siderophore loses two protons and forms a five-member ring structure with Fe (Kraemer, 2004). Bacterial species are the most dominant species for catecholate types of siderophore production (Dave et al., 2006). The common bacterial species are *Escherichia coli, Salmonella typhimurium*, and *Klebsiella pnemoniae* which dominantly produce enterochelin subtypes of catecholate types of siderophore production (Dertz et al., 2006). The bacterial species *Azotobacter vinelandii* is the source of various types of catecholate siderophores such as monocatecholate aminochelin, dicatecholate azotochelin, and tricatecholate protochelin under iron-limiting conditions (Wittmann et al., 2001).

Carboxyalate-type siderophore is a unique class of siderophore, which bears hydroxyl and carboxyl compounds (Dave and Dube, 2000); carboxyalate-type siderophore is neither related to hydroxamate nor phenolate ligands. Bacterial species such as Staphylococci, *Rhizobium meliloti*, and Mucorals are the sources of Staphyloferrin A and B, rhizobactin, and rhizoferrin carboxylate siderophores, respectively. Many siderophoral species such as lysine derivative, ornithine derivative, and histidine derivative contain mixed ligands with Fe^3+^ ions. Mycobactins are lysine derivative siderophores that bear 2-hydroxy phenyl oxazoline compounds which recover iron. Mycobactin siderophore is produced by *Mycobacteria* bacterial species, therefore, called mycobactin, which consists of two hydroxamate, one phenolate, and another oxazoline nitrogen. Pyoverdine is a dihydroxyquinoline compound, and structurally every pyoverdine siderophore differs from each other, while chromophore (1S)-5-amino-2,3-hydro-8,9-dihydro-8,9-dihydroxy-1H-pyrimido[1,2-a] quinoline-1 carboxylic acid shows similarities with azobactin that secretes by *A. vinelandii*. Pyoverdines and pseudobactins are isolated by *pseudomonas* bacterial species and are applicable in agriculture sectors and as human pathogens (Kloepper et al., 1980). The anguibactin siderophore is a histamine derivative mixed ligands siderophore; structurally anguibactin siderophore is a ω-N-hydroxy-ω-[[2′-(2",3"-dihydroxyphenyl) thiazolin-4′-yl]-carboxy] histamine. The anguibactin siderophore is applicable in living cells as an inducer for iron uptake.

## Action and Strategies of Siderophores

**Figure 3:**
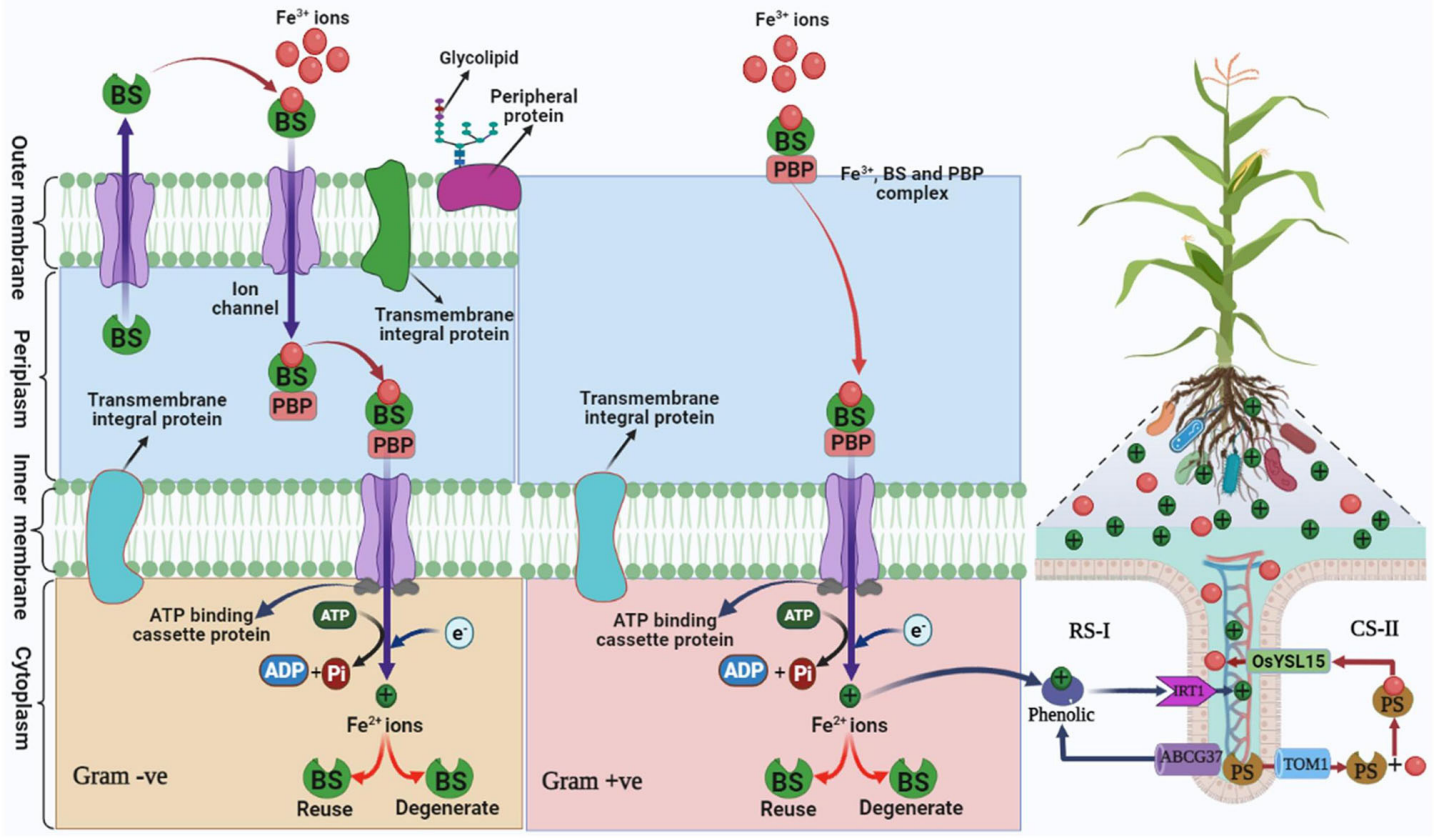
Mechanism of siderophore in plant growth-promoting gram-positive and gram-negative bacteria mediating iron uptake in plants under iron-deficient/degraded soil. Bacterial siderophore (BS), periplasmic binding protein (PBP), reduction strategy (RS-I), chelation strategy (CS-II), and plant siderophore (PS). Iron regulated transporter 1 (IRT), Yellow Stripe-Like Transporter of *Oryza sativa* (OsYSL15), ATP-binding cassette transporter (ABC) G37, translocase of outer membrane 1 (TOM) (Modified as sources of Fukushima et al., 2013; Seyoum et al., 2021).

Several microbes such as bacteria, fungi, algae, and dicotyledonous plants (Das et al., 2007) are involved in siderophoral activities that solubilize Fe^**3+**^ ions in the simple form and which are transported through specific receptos proteins in cells (Diaz de Villegas, 2007). This mechanism involves the reduction of a complex form of iron (Fe^3+^) to a simple form of iron (Fe^**2+**^) (Butler and Martin, 2005; Hopkinson and Morel, 2009). The transport systems of Fe-siderophore in Gram-negative and Gram-positive bacteria are different, the outer membrane transporters are broadly absent in Gram-positive bacteria, while they are found in Gram-negative bacteria and play an impressive role in the transport of Fe-siderophore. In Gram-negative bacteria, the Fe-siderophore passes on to the periplasmic binding protein-mediated TonB-ExbBD complex (Ferguson and Deisenhofer, 2002; Koebnik, 2005), and the bound Fe-siderophore with surface-binding-proteins are then imported into the cytoplasm *via* the possible siderophore-permease-ATPase system. The role of the surface periplasmic binding protein, ATPase, and permeases in Gram-positive bacteria is similar as in Gram-negative bacteria mediated by periplasmic surface binding protein permease with the ATP system (Fukushima et al., 2013). The movement of siderophore across the bacterial cell membrane owing to chemiosmotic potential is mediated by a complex of three membrane-spanning proteins (TonB, ExbD, and ExbB; Ferguson and Deisenhofer, 2002). TonB-dependent outer membrane receptors are involved in the adhesion of Fe^3+^ siderophore complexes on the bacterial cell surface (Schalk et al., 2012). Then Fe^3+^ siderophore complex is transported from outside of the outer membrane to the cell through the outer membrane of a bacterial cell by energy-dependent system and reaches the periplasm (Schalk et al., 2012). Afterward, Fe^3+^ siderophore complex ions bind with periplasmic binding protein (PBP) (Noinaj et al., 2010; Ribeiro and Simoe, 2019; Figure 3). Iron (Fe^3+^) siderophore complex is transported from the periplasm to the cytoplasm across the inner membrane by ATP binding cassette system and reaches the cytoplasm due to the reduction in Fe^3+^ ions to form Fe^**2+**^ ions. With this process being repeated in the bacterial cell, Fe^**+2**^ ions are directly absorbed by the rhizosphere of plants that promote the growth of plants (Ahmed and Holmstrom, 2014). In the case of gram-positive bacteria, due to the lack of an outer membrane all the processes occur in the periplasm and cytoplasm, Fe^3+^ siderophore complex ions adhere to the surface of periplasm, and the establishment of Fe^**2+**^ ions occurs in the cytoplasm (Faraldo-Gomez and Sansom, 2003; Fukushima et al., 2013; Schalk and Guillon, 2013; Ribeiro and Simoe, 2019; Figure 3).

Two strategies have been reported in the rhizospheric region to maintain iron uptake mediated by siderophore under iron-deficient soil. The first is a reduction strategy (RS-I), and second, a chelation strategy (CS-II) (Rai et al., 2021; Figure 3). Among both strategies, chelation strategies are common under stress conditions and tolerate a change in pH as compared to reduction strategy (Ahmed and Holmstrom, 2014), while in rice plants, both the strategies are reported (Krohling et al., 2016). RS-I is common in non-grass plants under low iron conditions in the rhizosphere where H^+^-ATPase AHA2 releases H^+^ and reduces the pH of the soil and induces the solubility of Fe^3+^. Iron once in apoplast gets chelated by phenolic compounds of the coumarin family and is transported by transporter ABCG37 (Mladenka et al., 2010). Ferric chelate reductase (Ferric Reduction Oxidase-2) reduces Fe^3+^-Fe^2+^ in the plasma membrane (Ahmed and Holmstrom, 2014). IRT1 (Iron Regulator Transporter-1) transports Fe^2+^ in the epidermal cell of plant root (Barberon et al., 2014).

Microorganisms such as bacteria/fungi and grasses follow the mechanism of CS-II. This strategy is commonly found in alkaline soil where acidification of rhizosphere is too difficult, thus bacteria are remarkable agents for their application in alkaline soil as well as stressed soil. This strategy (CS-II) is based on biosynthesis, secretion of siderophore such as phytosiderophore (PS)/bacterial siderophore (BS) that chelates Fe^3+^ and form Fe^3+^-BS/Fe^3+^-PS complex and transported through YS/YSL (Yellow Stripe/Yellow Stripe-Like) and TOM1 transporter family to the root (Dai et al., 2018).

## Genetic Mechanisms and Regulation of Siderophores

The key enzyme “non-ribosomal cytoplasmic synthase” produces siderophore by utilizing the precursors such as citrate, amino acids, dihydroxybenzoate, and N5-acyl-N5-hydroxyornithine, and their genes have been identified in several microorganisms (Paul et al., 2014; Paul and Dubey, 2015). In microbes such as bacteria, *Aspergillus fumigates*, yeast siderophore operon consists of several genes namely *sid*A, *sid*D, *sid*G, *sid*F, *sid*C and *sid*L which are located on different chromosomes (Blatzer et al., 2011; Khan et al., 2018; El-Maraghy et al., 2020).

The *sid*C gene, highly conserved among fungi, is characterized as non-ribosomal peptide synthetase (NRPS) and required for the biosynthesis of both ferricrocin (FC) and hydroxyferricrocin (HFC) (Schrettl et al., 2007). The *sid*F and *sid*G genes are characterized as acetyl transferase having the role of TAFC biosynthesis. The *sid*A gene encodes an L-ornithine N5-monooxygenase which initiates siderophore production (Seifert et al., 2008) whereas *sid*L gene which is located in cytoplasm and is a constitutively active N5-hydroxyornithine-acetylase required for FC biosynthesis (Blatzer et al., 2011). The siderophores uptake is facilitated by siderophore uptake genes i.e., *sit*1, *mir*B and *mir*C (Silva-Bailao et al., 2014). In contrast to fungi, the biosynthesis of different siderophores in bacteria has been governed by different genes such as *ent*B gene (enterobactin biosynthesis), *iro*B gene (salmochelin biosynthesis), *ent*S gene (enterobactin synthesis) (Watts et al., 2011; Paul and Dubey, 2015). In *E. coli*, the enterobactin synthesis operon consists of *ent*CDEBAH genes whereas, for enterobactin uptake and utilization, *fep*A, *fep*B, *fep*C, *fep*D, *fep*E, *fep*G, *fes*, and *ent*S genes are responsible (Peralta et al., 2016). In the gram-negative *Yersinia pestis* bacterium, the siderophore type yersiniabactin is synthesized by *irp*1 and *irp*2 genes (Guilvout et al., 1993). Etchegaray et al. (2004) reported that siderophores in *Xanthomonas* species are synthesized by non-ribosomal peptide synthetase from a precursor such as polyamine derivatives. Najimi et al. (2008) identified *asb*G, *asb*F, *asb*D, *asb*C, *asb*B, and *asb*I genes encoding proteins similar to components of the siderophore biosynthetic machinery in *Aeromonas salmonicida* bacteria. In *P. aeruginosa*, the siderophore pyochelin is synthesized by the genes *pchDCBA* and *pchEF*, and pyochelin precursors such as salicylate and dihydroaeruginoate (Dha), are clustered with the pyochelin regulatory genes *pchR* on its genome (Reimmann et al., 2001). Searle et al. (2015) developed multiple primers to screen environment samples for the presence of different microbial siderophores such as Enterobactin (*ent*A, *ent*B, *ent*C, *ent*E, *fep*A genes), Salmochelin (*iro*B, *iro*C, *iro*D, *iro*E, *iron* genes), Yersiniabactin (*irp*1, *irp*2, *irp*3, *irp*4, & 5, *fyu*A genes) and Aerobactin (*iuc*A, *iuc*B, *iuc*C, *iuc*D, *iut*A genes). Hofmann et al. (2020) reported that the gene *grdes*A from *Gordoniarub ripertincta* CWB2 and *psdes* A from *Pimelobacter simplex* VkMAC-2033D encodes lysine decarboxylases presumed to be involved in the synthesis of desferrioxamine siderophores. Wang et al. (2021) identified a novel Non-ribosomal Peptide Synthetase (NRPS) cluster in the bacteria *Burkholderia seminalis* strain R456 which is responsible for the production of a novel undescribed siderophore, along with previously reported ornibactin and pyochelin type siderophores, and also it is a crucial component in regulator protein Fur which regulates siderophore production.

## Sustainable Application of SPM For Plant Growth in Iron Deficient Degraded Land

Siderophore-producing microbes reduce the Fe deficiency and enhance all physiological and biochemical processes of crops in saline soil (Table 1). Siderophore-producing microbes *B. aryabhattai* MS3 are the most applicable in rice plants that enhance 60 and 43% of crop production under non-saline and saline (200 mM NaCl) conditions, respectively (Sultana et al., 2021). Siderophore-producing microbe *B. subtilis* DR2 act as a biofertilizer and promotes seed germination and plant growth in *Coriandrum sativum* (Kumari et al., 2021). Rangseekaew et al. (2021) reported that a specific bacterial strain of deep-sea *Dermacoccus barathri* MT2.1T and *D*. *profundi* MT2.2T strain have the ability to promote seedling in tomato plants under 150 mM concentration of NaCl as compared to the terrestrial strain *D. nishinomiyaensis* DSM20448T, due to the production of many plant-growth promoting attributes such as siderophore production, indole-3-acetic acid, and phosphate solubilization. Nadeem et al. (2012) reported that rhizospheric bacterial species *Variovorax paradoxus* (JN858091), *P. fluorescens* (JN858088), and *B. megeterium* (JN858098) have potential PGP attributes such as siderophore production, phosphate solubilization, exopolysaccharides production, indole acetic acid production, and ACC deaminase activity under both saline and normal conditions that alleviate the negative impacts of salinity and enhance the nutrients uptake for plant growth in cucumber plants.

Siderophore-producing microbes can produce plant growth-promoting attributes such as plant hormones, phosphate solubilization, secondary metabolites, etc., and provide suitable environments in stressed soil that enhances plant growth such as drought (Vivas et al., 2003; Breitkreuz et al., 2021). *B. subtilis* produce iron-chelating compounds that enhance the nutrient level in soil resulting in the growth of wheat plants under drought conditions (Lastochkina et al., 2020). Two siderophore-producing rhizobacterial species such as *P. putida* and *B. amyloliquefaciens* have the tolerance ability under drought stress due to the secretion of PGP attributes like siderophore production, hormone production, mineral solubilization, biofilm formation, and ACC deaminase activity, ameliorating the negative effects of drought and ensuring potential growth of *Cicer arietinum* L. under drought stress (Kumar et al., 2016). Several plant growth microbes survive under drought stress enhancing plant growth and yields; the inoculation of *Bacillus* sp. in lettuce increases the nitrogen, phosphorous, and potassium nutrients under drought stress conditions (Vivas et al., 2003). Siderophore-producing microbe *Pseudomonas* strains enhance the soil nutrients and other activities, including phosphate solubilization, potassium solubilization, and siderophore production under drought conditions (Breitkreuz et al., 2021).

Siderophore changes the oxidation states of heavy metals including Cd, Cu, Ni, Pb, Zn and Th^4+^, U^4+^, and Pu^4+^ to make them less toxic in nature (Schalk et al., 2011). Siderophores also bind different toxic metals such as Cr^3+^, Cu^3+^, Pb^2+^, Cu^2+^, V^4+^, and Al^3+^, while the binding capability of siderophores to Fe is more as compared to toxic heavy metals (Baysse et al., 2000; Braud et al., 2009b). Siderophores bind to toxic heavy metals, and thus toxic heavy metals do not hinder the efficiency of plant cells (Braud et al., 2009b). Therefore, the toxic heavy metal detoxifying and binding capability of siderophore plays a remarkable role in plant growth under heavy metal polluted land.

Siderophore has a strong affinity for the formation of iron-chelating compounds that help in the bioremediation process enhancing nutrient uptake and plant growth (Rajkumar et al., 2010). Bacterial strain *P. fluorescence* produces pyoverdine-type of siderophore that enhances mobility and reduces the toxicity of heavy metals in uranium mines (Edberg et al., 2010). Sharma and Johri (2003) reported that plant growth-promoting rhizobacterial genus *Pseudomonas* isolated from *Zea mays* L. secretes siderophores that have the potential to mobilize iron and have a high affinity to chelate Fe^3+^ ions resulting in heavy metals uptake. *Pseudomonas* strain GRP3 producing siderophore enhances the chlorophyll level in siderophore-treated mung bean plants (Sharma and Johri, 2003), and phytosiderophore enhances the iron efficiency of barley and wheat. Vishnupradeep et al. (2022) reported that two bacterial species *Providencia* sp. (TCR05) and *Proteus mirabilis* (TCR20) reduce the Cr toxicity from Cr(VI) to Cr(III) and enhanced plant pigments, protein, phenolics, and relative water content, while proline, lipid peroxidation, and superoxide dismutase decreased in *Zea mays* under heavy metal contaminated and drought conditions. Siderophore-producing microbes have the potential for phytoremediation of heavy metals and can overcome iron deficiency (Rajkumar et al., 2010). Dimkpa et al. (2009) reported that PGP rhizobacterial species *Streptomyces tendae* F4 phytoremediated cadmium (Cd) and enhanced the uptake of metals in heavy metals polluted lands. *Enterobacter cloacae* rhizospheric bacteria isolated from *Spilanthes acmella* Murr (toothache plant) of Shivalik hills region secretes PGP attributes including, exopolysaccharides (EPS) and 1-aminocyclopropane-a-carboxylic acid (ACC), acts as a biocontrol and biofertilizer under drought stress conditions (Thakur et al., 2021). Symbiotic association among plant and SPM is potentially involved in heavy metal uptake, SPM *Rhizobium* strains promoted Cu uptake while *Pseudomonas* strain promoted Cu and Fe uptake by *Phaseolus vulgaris* plants (Carrillo-Castaneda et al., 2007), and *S. acidiscabies* SPM secretes hydroxamate types of siderophores responsible for the solubilization and uptake of nickel and iron by *Vigna unguiculata* plants under nickel stress condition (Dimkpa et al., 2008). Symbiotic association of SPM *Kluyvera ascorbata* and plants decreased the toxicity of heavy metals (Burd et al., 2000) and suppressed the phytopathogens (Glick, 2012).

Siderophores maintain iron starvation in plants (Sayyed et al., 2019) and suppress the phytopathogens (Shaikh et al., 2014; Saha et al., 2016; Sayyed et al., 2019) like *Phytophthora parasitica* (Seuk et al., 1988), *Phythium Ultimum* (Hamdan et al., 1991). Ghazy and El-Nahrawy (2021) reported that bacterial strains such as *B. subtilis* MF497446 and *P. koreensis* MG209738 produce siderophores and induce disease resistance against *Cephalosporium maydis* in maize crops. *Brevibacillus brevis* GZDF3 (PGPR strain) isolated from the rhizosphere of *Pinellia ternate* plants play an important role in antagonistic activity against *Candida albicans* fungal disease by siderophore production (Mohammed et al., 2020; Sheng et al., 2020); *P. flurescens* and *P. aeruginosa* bacterial strain act as a biocontrol agent against *Ralstonia solanacerum* of tomato wilt. Siderophore-producing microbes, namely gram-negative bacteria *Escherichia coli*, secretes secondary metabolites such as siderophores that enhance iron uptake and plant growth performances under iron stress conditions (Neilands, 1995), and gram-negative bacterial genus *Streptomyces* acts as a biofertilizer that enhances the plant nutrients (Fe, P, and N), significantly increasing the germination rate, shoot length, and dry weight of wheat plant under saline stress condition (Sadeghi et al., 2012; Upadhyay et al., 2019).

## Conclusion and Way Forwards

A proportional relation exists between growth performance and yield of plants; however, a big challenge arises in this proportional relationship due to the rapid rise in degraded land across the globe. The utilization of degraded land for agricultural practices becomes an issue for researchers to meet global food production for the future with eco-friendly and sustainable technology. Degraded land poses several detrimental impacts on plant growth and induces plant stress by less cycling of available nutrients and disruption in the metabolic function of the plant. The review discussed the influence of iron-deficient soil on plant and their management through eco-friendly products i.e., siderophores. The diverse chemical nature of siderophores can chelate Fe^3+^, which is produced by siderophore-producing rhizobacteria, and plant roots commonly known as bacterial siderophore (BS) and plant siderophore (PS).

In the rhizospheric microenvironment, both BS and PS synergistically facilitate iron uptake in the plant from iron-deficient soil mediated by reduction and chelation strategies. The utilization of siderophore-producing rhizobacteria can effectively maintain the iron level in plants and induce plant growth performances under degraded soil effectively when their selections meet compatibly with plant roots specifically. Future research requires the selection of the perfect candidate for siderophore-producing rhizobacteria, for a specific plant in degraded soil that would be useful for plant stress management and plant productivity at the field level.

## Author Contributions

SU, PC, DJ, PS, and RS: conceptualization and visualization of the present review and writing the original draft. SU and PC: prepared the figures and tables. DJ, PD, and RS: contributed to re-structuring the review. PD, JW, and MJ contributed special remarks and edited the review for final submission. All authors contributed to the article and approved the submitted version.

## Funding

The authors acknowledge the funding for the Science and Technology Major Project of Guangxi (AA18242026) and National Natural Science Foundation of China (81960164).

## Conflict of Interest

The authors declare that the research was conducted in the absence of any commercial or financial relationships that could be construed as a potential conflict of interest.

## Publisher's Note

All claims expressed in this article are solely those of the authors and do not necessarily represent those of their affiliated organizations, or those of the publisher, the editors and the reviewers. Any product that may be evaluated in this article, or claim that may be made by its manufacturer, is not guaranteed or endorsed by the publisher.
